# Advancing the evaluation of integrated knowledge translation

**DOI:** 10.1186/s12961-018-0383-0

**Published:** 2018-11-06

**Authors:** Sara A. Kreindler

**Affiliations:** 1 Department of Community Health Sciences, University of Manitoba, 451-753 McDermot Ave., Winnipeg, MB R3E 0T6 Canada; 2 George & Fay Yee Centre for Healthcare Innovation, University of Manitoba, 451-753 McDermot Ave, Winnipeg, MB R3E 0T6 Canada

**Keywords:** Knowledge translation, Realistic evaluation, Evidence-informed decision-making, Research methods

## Abstract

**Background:**

Integrated knowledge translation (IKT) flows from the premise that knowledge co-produced with decision-makers is more likely to inform subsequent decisions. However, evaluations of manager/policy-maker-focused IKT often concentrate on intermediate outcomes, stopping short of assessing whether research findings have contributed to identifiable organisational action. Such hesitancy may reflect the difficulty of tracing the causes of this distal, multifactorial outcome. This paper elucidates how an approach based on realistic evaluation could advance the field.

**Main Text:**

Realistic evaluation views outcomes as a joint product of intervention mechanisms and context. Through identification of context–mechanism–outcome configurations, it enables the systematic testing and refinement of ‘mid-range theory’ applicable to diverse interventions that share a similar underlying logic of action. The ‘context-sensitive causal chain’ diagram, a tool adapted from the broader theory-based evaluation literature, offers a useful means of visualising the posited chain from activities to outcomes via mechanisms, and the context factors that facilitate or disrupt each linkage (e.g. activity–mechanism, mechanism–outcome).

Drawing on relevant literature, this paper proposes a context-sensitive causal chain by which IKT may generate instrumental use of research findings (i.e. direct use to make a concrete decision) and identifies an existing tool to assess this outcome, then adapts the chain to describe a more subtle, indirect pathway of influence. Key mechanisms include capacity- and relationship-building among researchers and decision-makers, changes in the (perceived) credibility and usability of findings, changes in decision-makers’ beliefs and attitudes, and incorporation of new knowledge in an actual decision. Project-specific context factors may impinge upon each linkage; equally important is the organisation’s absorptive capacity, namely its overall ability to acquire, assimilate and apply knowledge. Given a sufficiently poor decision-making environment, even well-implemented IKT that triggers important mechanisms may fall short of its desired outcomes. Further research may identify additional mechanisms and context factors.

**Conclusion:**

By investigating ‘what it is about an intervention that works, for whom, under what conditions’, realistic evaluation addresses questions of causality head-on without sacrificing complexity. A realist approach could contribute greatly to our ability to assess – and, ultimately, to increase – the value of IKT.

## Background

Integrated knowledge translation (IKT) has been defined as “*a model of collaborative research*” in which “*researchers work with knowledge users who identify a problem and have the authority to implement the research recommendations*” ([1], p. 299). This approach, also known as ‘engaged scholarship’, emerged from the realisation that, despite researchers’ efforts to ‘transfer’ knowledge to decision-makers, research findings were not in fact being implemented. In a seminal article, Van de Ven and Johnson reframed the knowledge–practice gap as a problem of knowledge production rather than knowledge transfer – if, they argued, decision-makers were engaged in co-producing knowledge relevant to their own practice, they would convert this knowledge into “*actions that address problems of what to do in a given domain*” ([2], p. 803). The idea of converting knowledge into ‘actions’ or ‘solutions’ remains fundamental to IKT [3, 4]. The foundational premise of IKT is that active engagement in the process of knowledge production increases decision-makers’ propensity to use the resultant knowledge, not merely for conceptual enlightenment (conceptual use), nor to legitimise decisions already made (symbolic use), but to inform tangible actions (instrumental use; see [5, 6]). If this premise is accurate, we should expect to find an association between IKT and instrumental use of research; where IKT is undertaken with managers or policy-makers, it should increase the likelihood that organisational actions will reflect research findings.

Surprisingly, however, a recent scoping review found that, out of 13 IKT studies, only 4 assessed whether any influence on policy or service delivery had occurred (of these, 2 reported inconclusive findings) [7]. Much more commonly mentioned were intermediate outcomes such as capacity development on the part of researchers and decision-makers, improved intergroup attitudes or relationships, and enhanced research relevance. Even in the broader literature on organisational knowledge translation (KT; of which organisational IKT is a subset), few studies have assessed whether the organisation’s eventual decision is congruent with evidence – indeed, some scholars hold it to be unrealistic for researchers even to aspire to such an outcome [8]. However, if we want to know whether, or under what conditions, IKT can remedy the problem it was designed to address, then the question of whether findings contribute to organisational action must be a major focus of evaluation.

It is frequently argued that organisational decision-making is too complex and multifactorial for easy attributions of causality, and that KT may produce subtle, long-term influence even in the absence of immediate, tangible impacts. Yet, this is also true of other health-services and policy interventions that we do subject to rigorous outcome evaluation. IKT is a complex intervention implemented in a complex context; such interventions are poor candidates for so-called ‘black-box’ evaluations (i.e. those that merely assess outcomes, leaving causal processes opaque), but much can be gained from a theory-based approach that can trace and explicate the process by which the intervention produces its outcomes. This paper proposes that realistic evaluation [9] is ideally suited to IKT evaluation; its intent is to provide guidance for conceptualising an evaluation of IKT through a realist lens.

This paper first provides an overview of realistic evaluation, acknowledges some of its challenges, and suggests a technique that can mitigate these challenges. Second, it applies realist concepts to the IKT field, delineating a theory of how IKT may lead to instrumental use of research findings. Finally, it comments on implications for further research.

## Main text

### Realistic evaluation

Realistic evaluation takes its name from the paradigm of scientific realism, which is concerned with the identification and understanding of causal mechanisms [9, 10]. Its classic evaluation question is ‘What is it about this intervention that works, for whom, and under what conditions?’ Realistic evaluation recognises that an attempt to answer this complex question through an inductive search for associations among myriad intervention features and context factors would be not merely exhausting but ultimately uninformative. Instead, its approach is to generate and test theory on the mechanism(s) by which the intervention produces its effects, and key contextual elements required for their operation. Like all members of the theory-based evaluation family, realistic evaluation is grounded in the insight that every intervention reflects a ‘programme theory’, a posited causal chain from activities to outcomes via mechanisms; the focus on mechanisms rather than intervention activities allows the development of mid-range theory applicable to diverse interventions that share a similar underlying logic of action [11, 12]. Unlike earlier approaches, realistic evaluation examines outcomes as a joint product of mechanisms and context, and focuses on the identification of context–mechanism–outcome (CMO) configurations [12]. Context factors dictate the ‘scope conditions’ of the programme theory, that is, the conditions under which it will or will not operate [13].

It must be emphasised that mechanisms are not intervention components/activities; they are system responses triggered by the intervention that, in turn, generate outcomes [9, 11]. This sequence may be represented as follows: I (intervention) ➔ M (mechanism) ➔ O (outcome). ‘Context’ refers to features of the system that may impinge on these linkages. Several articles have sought to elucidate the frequently misunderstood concept of mechanism and its relationship to context [14–16]. My personal heuristic is to use the word ‘because’ for mechanisms (‘the intervention works because it triggers this response…’) and ‘unless’ for context factors (‘the intervention will/will not work unless these conditions are present…’). In other words, a ‘because’ factor (mechanism) is something brought about by the intervention that is key to its effectiveness; an ‘unless’ factor (context) is something external to the intervention that enables or inhibits its effectiveness (sometimes an apparent ‘unless’ factor may turn out to be an intervention flaw or a countervailing mechanism rather than a context factor per se; such issues can be clarified after the initial because/unless distinction).

Interest in realistic evaluation has exploded; the number of realistic evaluations of KT continues to grow (although, to my knowledge, there has been only one of IKT) [17, 18]. However, the development of mid-range theory can be fraught with difficulties, particularly for complex interventions involving multiple mechanisms [17]. If CMO configurations are defined too narrowly or too broadly, the evaluator risks either drowning in detail or articulating propositions too vague to have real explanatory power. Furthermore, evaluators are wont to generate CMO configurations in a piecemeal and idiosyncratic manner; as a result, rather than cumulate, a series of evaluations may produce a plethora of theoretical statements that defy synthesis. In order to overcome such challenges, it may be useful to rediscover a tool from the original theory-based evaluation toolkit – the causal chain. Weiss’ foundational work on theory-based evaluation recommended that evaluators map out the cascading chain of mechanisms whereby a programme is expected to achieve its effects [11]. This visual aid makes the programme theory explicit, making it easier to test whether each of the posited linkages actually occurs as predicted. The ‘causal chain’ technique appears to have fallen out of favour as a result of misuse – the field saw too many ‘logic models’ populated with sequences of activities instead of mechanisms, haphazardly defined categories, or blanket terms that left most of the causal processes obscure [14]. Used correctly, however, a causal chain diagram can be highly useful for delineating a programme theory, or several alternative theories, in the form I ➔ M1 ➔ M2 ➔ M3…➔ O. It is important to note that the presentation of a simple, linear causal chain implies no assumption that the intervention’s workings are simple and linear in the real world; on the contrary, the purpose of a causal chain diagram is to enable a structured inquiry into how and where these workings depart from simplicity and linearity.

**Fig. 1:**
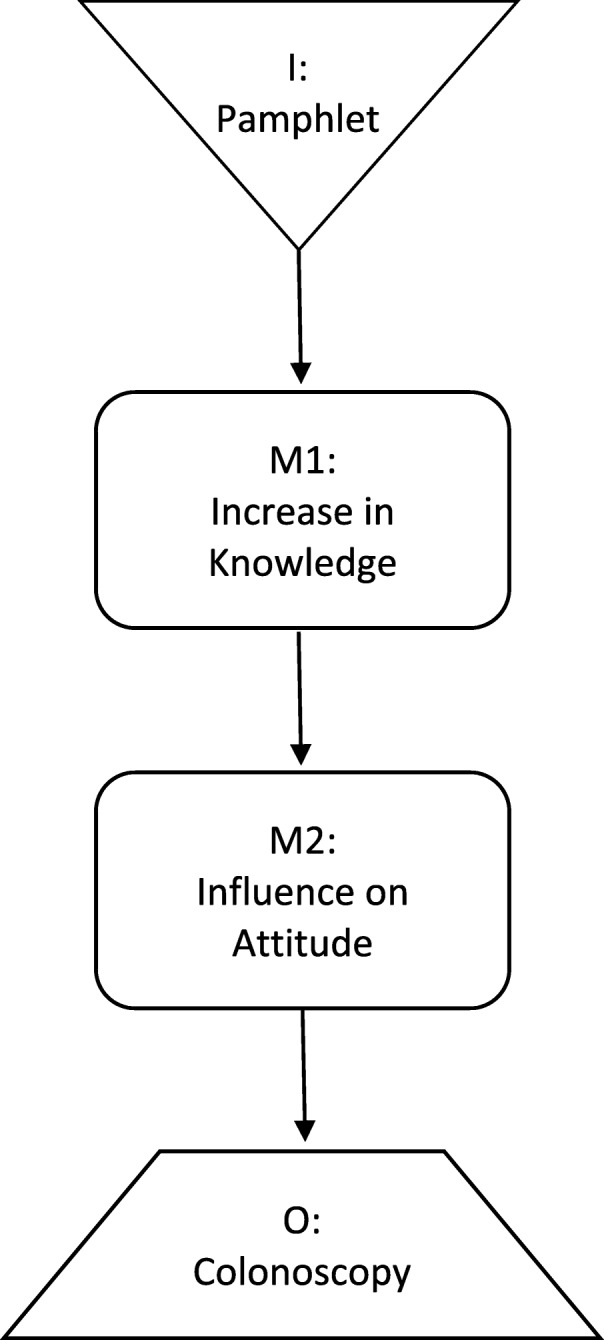
Example of a causal chain

To illustrate causal chains and how they can be used in realistic evaluation, this paper will use a very simple, non-IKT intervention as an example. A pamphlet about colon cancer (I) might be intended to increase patient knowledge (M1), hence improving their attitudes towards screening (M2), resulting in their presentation for a colonoscopy (O) (Fig. 1). Even in this simple example, the development of a causal chain diagram demands numerous decisions. First, one must decide how proximal or distal an outcome should be defined as the endpoint of the causal chain (O). The pamphlet’s manifest objective is patient presentation for colonoscopy; however, it could be argued that its ultimate goal is the improvement of health outcomes via early detection of colon cancer. It could further be argued that increased uptake of colonoscopy does not inevitably produce better patient and system outcomes, but may instead lead to unnecessary testing, causing queues, waste and distress. Thus, the causal chain might be extended ad infinitum to explore all potential downstream impacts of the intervention. However, Fig. 1 reflects the view that questions about the ultimate ramifications of screening are important when evaluating screening, but not necessarily when evaluating pamphlets; we can learn just as much about how pamphlets work as a communication tool while leaving their downstream impacts out of scope. Should evaluators wish to explore more distal outcomes, additional layer(s) can be appended to the causal chain (O1, O2, etc.) (if multiple unrelated outcomes are of interest, it is preferable to draw separate causal chains than to allow the diagram to become unwieldy). Second, one must decide to what extent to ‘lump’ or ‘split’ mechanisms. Treating every possible variant of a mechanism separately will make the model unmanageably complex; conflating mechanisms that operate through distinct causal pathways will impair the model’s explanatory power [11]. A sensible guiding principle is that, if each sub-element of M1 can be anticipated to trigger each sub-element of M2, then the sub-elements within each mechanism can be treated as ‘interchangeable parts’ unless proven otherwise.

The landmark contribution of realistic evaluation to theory-based evaluation is the concept of the CMO configuration; that is, the idea that the production of a given outcome requires both an appropriate mechanism and a facilitative context. We can integrate this concept with the causal-chain technique by observing that different context factors are relevant at different points along a causal chain; they may disrupt or facilitate any of the linkages between intervention, mechanism(s) and outcome. I personally find it more intuitive to frame context factors as barriers/disruptors than as facilitators/enablers, as this allows me to use an ‘it will work unless’ formulation and visualise a broken strand of wiring. However, barriers and facilitators are mirror images of each other (generally speaking, if X is a facilitator, then lack of X is a barrier, and vice versa), so some may prefer to use an ‘it will not work unless’ formulation and visualise insulation on a segment of wiring. The important part is to attach each context factor to the specific linkage(s) it is posited to influence; otherwise, the concept of CMO configuration is lost [19].

**Fig. 2:**
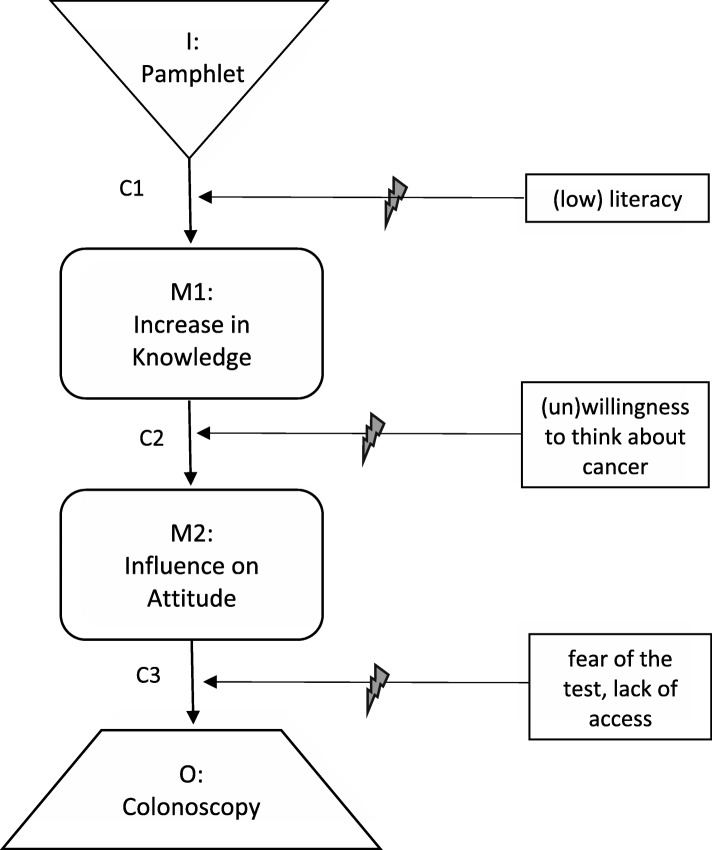
Example of a context-sensitive causal chain

Figure 2 transforms Fig. 1 into a ‘context-sensitive causal chain’ by specifying which link(s) are affected by different context factors, using the lightning bolt symbol to indicate potential disruption. For example, the pamphlet may fail to generate knowledge (I–M1) among patients with low literacy (C1); knowledge may not spark attitude change (M1–M2) in patients averse to thinking about cancer (C2); and attitudes that are positive in theory may not translate into behaviour (M2–O) among patients who fear the discomfort of a colonoscopy or lack access to the service (C3). It is advisable to restrict each causal chain to one outcome (or the diagram becomes too complex to facilitate clear conceptualisation), but separate causal chains can be drawn for multiple outcomes, including unintended ones.

### Preliminary programme theories of IKT

#### A starting point

An early step in realistic evaluation is to articulate one or more testable programme theories based on what is already known or believed about the intervention [9]. The following section will do so for IKT, drawing on the literature. For this purpose, I will define IKT as ‘the’ intervention, making no attempt to specify which components might be associated with which mechanisms or outcomes. It should nonetheless be noted that IKT typically includes multiple components (e.g. interactive activities such as one-on-one or group meetings, teleconferences, e-mail discussion; formal or informal processes for collective decision-making; communication strategies such as using policy-maker-friendly language), which may vary [7]. The ‘project’ through which researchers and decision-makers co-produce knowledge may be any type of primary research or knowledge synthesis.

#### Defining the outcome

**Fig. 3:**
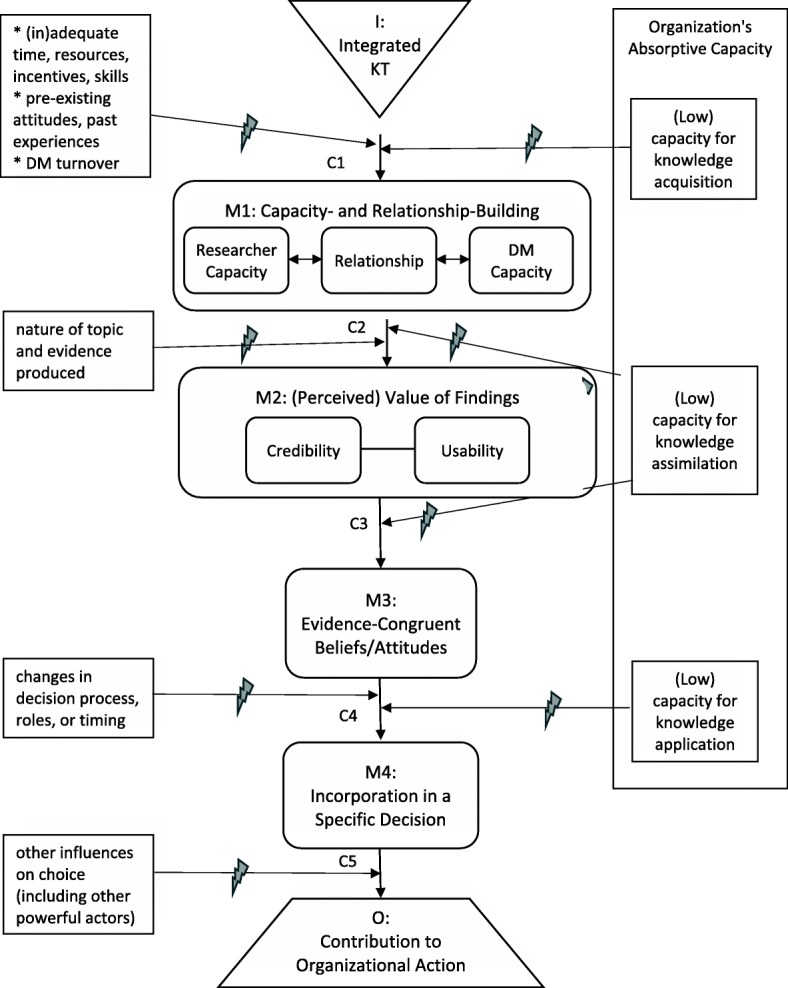
Causal chain linking IKT to evidence-informed organisational action (instrumental use of knowledge). *I* intervention, *M* mechanism, *C* context factor, *O* outcome, *DM* decision-maker

This paper began by suggesting that the defining aim of organisational IKT is to promote organisational action that reflects research findings, or instrumental use of research. As IKT is “*action-oriented and solutions-focused*” [3], IKT-based projects should strive to generate findings that can contribute to tangible decision(s) to adopt, avoid, modify or discontinue some type of policy, service or practice (all of which count as organisational action). However, as projects may vary widely in terms of the scope and urgency of the problems they address, it is important to define instrumental use as broadly as possible without losing its essence. The ‘O’ box in Fig. 3 should be understood to include any organisational action or decision to which research findings have contributed to any extent, at any time following the research; evaluators should ensure a sufficiently long timeframe to capture relevant organisational decisions, bearing in mind that the process of decision-making may be slow. However, the defined outcome excludes cases in which decision-makers discuss the findings but take no identifiable action consistent with them, or make their decision prior to consideration of the findings.

Some recent work from the related domain of patient/public involvement may provide a useful starting point for the assessment of this outcome. The authors of the Scoresheet for Tangible Effects of Patient Participation (STEPP), a tool to measure the instrumental use of patient input, began by recognising that instrumental use has two distinct components, namely the organisation (1) takes action that is in accordance with patient/public input, and (2) would not have taken identical action in the absence of this input [20]. To assess component 1 (‘organisation’s response’), they determined whether the organisation had taken action that was wholly or partially congruent with each patient-generated recommendation (in their tool, consideration of a recommendation counts as an action, but receives a lower score than partial or full implementation thereof). To assess component 2 (‘patient influence’), they asked decision-makers about the reasons for each action, and the extent to which patient input had been a contributing factor. It ensued that decision-makers readily differentiated among actions that had been determined, informed, confirmed or unaffected by patient input. Notwithstanding the well-known limitations of self-report, there appeared to be value in simply asking decision-makers what had contributed to their actions – so long as the questions addressed specific actions (e.g. ‘Did you do X?’ ‘Was this input the reason, or a reason, why you did X?’), and not merely the global matter of whether patient input had been ‘used’. Other methods of gauging influence (e.g. through document analysis) should also be explored.

To use the STEPP, a score is given to each patient-generated recommendation or issue for magnitude (size or importance), organisation’s response and patient influence, and these three scores are multiplied to produce a score for each recommendation/issue, which can then be combined into several types of composite scores [20]. As the STEPP has not been validated (beyond the preliminary validation undertaken during its four-site pilot study), it would be premature to recommend utilisation of its quantitative-scoring functionality. However, the tool does offer a useful structure for collecting and organising data, which can easily be applied to investigating the instrumental use of research findings. Of course, overall evaluation would go beyond assessment of decision-making outcomes, and would require the organisation to participate in ways beyond providing data on such outcomes (e.g. giving feedback on the collaborative process and the research itself).

#### Proposed mechanisms and corresponding context factors

What do we know about how IKT may lead to instrumental use? As realistic evaluation is concerned with mid-range theory, it is not necessary to restrict our inquiry to IKT. The mechanisms that underpin IKT are probably relevant to most interactive efforts to promote an organisation’s instrumental use of knowledge – that is, even if the interactive approach is less than ‘integrated’ (e.g. decision-makers’ participation is limited, or occurs through an intermediary such as a knowledge broker) or the knowledge is not research (e.g. evaluation, decision support, performance data, consultation findings). On the other hand, different mechanisms may be relevant to pure ‘push’ or ‘pull’ strategies, and to IKT oriented towards clinicians, patients or communities. The programme theory outlined below draws on reviews and conceptual papers about IKT, participatory or collaborative evaluation, organisational use of research or evaluation findings, and evidence-informed decision-making in general, in healthcare and (to a lesser extent) other public-sector contexts. However, as no comprehensive review of these literatures was attempted, it must be recognised as preliminary.

According to the theory delineated in Fig. 3, researcher–decision-maker collaboration (the intervention that is IKT) fosters both capacity-building and relationship-building (M1) [2, 18, 21–23]. Researchers gain skills in working and communicating effectively with decision-makers, and learn from them about organisational needs and context (M1a); decision-makers improve their research literacy and ability to work with researchers (M1b). The relationship between the two groups also develops or improves (M1c); this may involve such sub-mechanisms as trust, attitude change, erosion of intergroup boundaries, etc.

Capacity-building and relationship-building strengthen each other in a virtuous cycle, and each may increase the actual and/or perceived quality or value of research findings (M2) [2, 24–28]. Informed by Weiss and Bucuvulas’ finding that decision-makers subject information to distinct ‘truth tests’ and ‘utility tests’ [24], Fig. 3 presents the credibility and usability of findings as separate dimensions of (perceived) value. The knowledge, skills and relationships nurtured by IKT may increase both credibility (M2a; actual and perceived research quality, perception of the researcher as a trusted source, sense of ownership, etc.) and usability (M2b; actual and perceived relevance to decision-makers’ information needs, timeliness, action orientation, intelligibility, etc.) [25–28].

Information that decision-makers deem sufficiently credible and usable will trigger changes in their issue-related beliefs and attitudes (M3, which includes the acquisition, alteration, solidification or increased subjective importance of certain belief(s) or attitude(s)). Such changes may be mediated by various social-cognitive processes at the individual level, and may be amplified by processes of social influence at the interpersonal and group levels [26]. The more influence occurs, the greater the likelihood that the information will be incorporated in an actual decision-making opportunity (M4), which is a prerequisite for instrumental use (the term ‘incorporated’ is used to stress that the information is considered during decision-making, not merely contemplated in the abstract).

As with all causal chains, it is possible that countervailing mechanisms (e.g. co-optation of one party by another) will redirect the route so that its destination is other than the desired outcome (e.g. distortion or suppression of evidence) [14]. Such possibilities, while not detailed here, would also be appropriate areas of inquiry for realistic evaluation.

For ease of presentation, all the context factors discussed below are framed as barriers; as noted earlier, this framing can be reversed by describing the opposite of each factor as a facilitator/enabler. Of the contextual barriers that impinge upon the mechanisms of IKT, some relate to the issues, participants or constraints involved in a specific project. Challenges such as inadequate time or resources, lack of skill or sincerity on the part of researcher or decision-maker participants, negative past experiences, hostile intergroup attitudes, or turnover of decision-makers can impede the intervention from stimulating capacity- and relationship-building (C1) – or indeed, from even getting off the ground [7, 18, 29, 30]. Findings discordant with decision-makers’ expectations, values or experience may not be viewed as credible (C2a), while findings of exploratory, conceptual or simply inconclusive research may not be viewed as immediately usable (C2b) [24, 27, 28, 31]. Changes in the nature or timing of decisions to be made, or in decision-making roles, may impede findings from reaching a decision opportunity (C3; this includes cases in which a decision is made before the research is complete, or even before it begins). Finally, findings that are carefully weighed during decision-making may still not generate instrumental use if other considerations carry more weight, or if action is blocked by external forces; such barriers are especially likely to arise when the issue is highly politicised (C4) [22, 32].

While much of the existing IKT literature focuses on such project-specific factors, it is equally important to examine the overall organisational context in which IKT is attempted [33, 34]. Perhaps the most crucial aspect of organisational context is the degree to which the organisation is able to acquire, assimilate and act upon new knowledge – a tripartite capability known as absorptive capacity [35, 36]. In an organisation with low capacity for exploratory learning – one that does not value or support knowledge acquisition – researchers may struggle to even implement IKT, let alone to foster strong researcher–decision-maker partnerships and robust decision-maker capacity. In an organisation with low capacity for transformative learning – one in which a culture of fear, chronic lack of reflective time, absence of routines for knowledge sharing, or managerial incompetence impede the assimilation of new knowledge – evidence may have little influence, in part because it fails to be recognised as usable and/or credible [37].

If an organisation has low capacity for knowledge application or exploitative learning – if decision-making is typically a crisis-driven exercise in ‘jumping to solutions’ – then evidence that is readily assimilated by decision-makers still may not find its way into an actual decision [38]. Another useful construct for thinking about knowledge application is ‘procedural rationality’, namely the extent to which relevant information is brought into, and relied upon, during the decision-making process [39]. The hallmark of procedural rationality is the ‘discovery’ approach, in which multiple options are sought out, then evaluated; discovery contrasts with ‘idea imposition’, in which only one option is considered [40]. Research on organisational decision-making has confirmed that a discovery approach yields superior outcomes across a wide range of conditions, but is practised in only about one-third of strategic decisions [40–42]. Procedural irrationality is endemic to organisations that are ‘anarchic’, i.e. lacking in shared preferences, clear processes and consistent decision roles [43]. In such a poor decision-making environment, even skilful IKT efforts that trigger intermediate mechanisms may fall short of their desired outcomes [38].

One advantage of the realist approach is that it enables a more nuanced appraisal of intervention success or failure than mere assessment of whether instrumental use was produced. For example, if research findings are incorporated in managers’ deliberations (M4) but trumped by other important considerations (e.g. conflicting evidence, patient preferences; C5), an evaluator might deem the IKT enterprise successful even though no instrumental use occurred. On the other hand, if managers give the appearance of deliberating on research evidence but invariably decide to continue past practice, or if they invoke research during abstract discussions (M3) but never connect this to an actual decision opportunity (M4), an evaluator might infer that further, similar IKT activities in the same organisational context are unlikely to ensue in instrumental use. By following the causal chain all the way to instrumental use, the evaluator is better able to distinguish among such disparate situations and draw conclusions accordingly.

#### Other potential causal chains

The causal chain in Fig. 3 is a representation of only one plausible theory of how IKT may promote organisational action that reflects research findings. An alternative theory might suggest that research findings slowly percolate through an organisation, gradually entering into common parlance (conceptual use) and being promulgated by sympathetic advocates (symbolic use) [32] before some manager – who may or may not have been involved in the original research – finally applies them. Findings initially too controversial to be adopted might, via this pathway, eventually find their way to instrumental use. This programme theory would require a modified causal chain diagram, with an intervening mechanism between M2 and M3 (i.e. the credible, usable co-produced knowledge begins to percolate, even if some of its co-producers remain hostile to it), as well as a stipulation that all subsequent mechanisms may apply to decision-makers outside the research team. It would also require a longer evaluation timeframe than the more direct pathway outlined in Fig. 3. However, it can certainly be accommodated within a realist approach, as can other alternative theories.

### Considerations and implications

As discussed in the ‘colonoscopy pamphlet’ example, an intervention’s defining aim may not be its ultimate goal. The ultimate goal of IKT goes beyond instrumental use of co-produced knowledge – it is to foster decisions that lead to better outcomes for patients and the health system. In some cases, instrumental use of knowledge from a particular research project might not further this goal since the findings may be incongruent with other evidence, values or patient preferences (see C5 in Fig. 3); moreover, a policy that reflects the best available evidence may still fail to produce its anticipated benefits, or may benefit some at the expense of others. Furthermore, it is conceivable that IKT efforts might further the goal indirectly without achieving instrumental use – for instance, by increasing the organisation’s absorptive capacity (perhaps through the mechanism of conceptual use), increasing, in turn, the likelihood that future decisions will be evidence informed [44]. Whether to develop a causal chain diagram that includes the ultimate goal is left to the evaluator’s discretion. Though much could be learned about the benefits and limitations of evidence-informed decision-making by tracing the path from use (or non-use) of findings to patient and system outcomes, much can still be learned about how IKT works (or does not) without this additional step. The only circumstance in which I would recommend incorporating distal outcomes is when an evaluator suspects that IKT is producing those outcomes through a pathway that does not include instrumental use. Nonetheless, it should be noted that such potential pathways are quite distant from the programme theory implied within the main conceptual papers on IKT [1–4].

The attempt to articulate intelligible programme theories necessarily involves simplification; accordingly, I have intentionally limited the number of mechanisms, context factors and bidirectional arrows in the two figures. Most, if not all, of the posited mechanisms could be disaggregated; one might, for instance, distinguish among different domains of capacity-building, aspects of credibility or psychosocial processes underpinning attitude change [26]. All of the context factors could be unpacked to reveal additional layers; for example, one might identify elements of the organisational and external context that affect absorptive capacity [36]. Arrows or arrowheads might be added to express the cyclic or iterative nature of knowledge-to-action processes [45]. As a theory is refined through further research, mechanisms and context factors should be unpacked wherever the causal chain is found to break down and the cause of this breakdown cannot be well explained at the current level of analysis; reciprocal relationships should be specified wherever it becomes apparent that a feedback loop [46] is a principal cause of a key mechanism or outcome. Beyond this, however, the benefits of increased nuance must be weighed against the risks of excessive detail [11]. There is a limit to the amount of complexity a theory can accommodate before it mutates into a taxonomy or framework and causal relationships sink back into obscurity.

When the IKT literature is considered in light of the programme theory outlined above, it becomes evident that some segments of the posited causal chain have attracted more research than others. The greatest focus has been on the path from I to M1 (capacity- and relationship-building) [7, 18]. This stands to reason, as this mechanism is what most distinguishes IKT from other KT approaches, and addresses the most commonly identified barriers to evidence use [30]. However, once the most conspicuous barriers have been overcome, others may surface, some of which may reflect deep problems in an organisation’s decision-making culture [37]. A fuller investigation of such barriers could help KT practitioners make informed decisions about where and with whom to involve themselves, and may even reveal ways to adapt IKT to an inhospitable decision-making climate. Having started my career as an embedded researcher, I have been inclined to define the ideal model of IKT as deep researcher engagement in the overall process of addressing a complex, system-level, decision-maker-identified problem. However, it is possible that deep engagement in an anarchic organisation will more frequently lead to enmeshment in dysfunctional decision-making processes than to knowledge use, and that more impact could be achieved through limited collaborations on circumscribed issues. Research examining how the quality of a decision-making environment moderates the relationship between depth of researcher–organisation engagement and outcome achievement could provide important guidance to engaged scholars. To maximise learning, future studies could hone in on the least-understood areas of the causal chain; this might entail deliberately introducing skilled IKT practitioners into contexts that are favourable in some ways (e.g. C1) but unfavourable in others (e.g. C3 or especially C4). Further research may also identify additional mechanisms or context factors that are crucial to understanding the outcomes of IKT.

A realist approach that explicitly tests a preliminary programme theory offers several advantages over conventional evaluation approaches. First, it offers a systematic way to study the entire causal pathway between the intervention and its desired outcomes, permitting evaluators to assess the impacts of IKT fairly without confining their inquiry to proximal outcomes. Second, it helps evaluators to move beyond merely listing contextual barriers/facilitators to actually identifying where each is located on the causal chain. Third, it enables evaluations to cumulate by contributing to the testing of a common theory (or competing theories) [9]; thus far, evaluations of IKT have been too heterogeneous for their findings to be easily synthesised [7].

## Conclusions

To those grappling with the complexities of evaluating organisational IKT, and in particular gauging its contribution to evidence-informed action, realistic evaluation offers an approach that is both practical and conceptually sound. This paper has sought to encourage researchers to embrace this approach, to use context-sensitive causal chains as a tool for making mechanism–context interactions more intelligible, and to consider adopting the preliminary causal chain sketched out here as a starting point. The more clearly we can understand the complex journey from IKT to evidence-informed action, the better equipped we will be to design interventions that reach their intended destination.

## Abbreviations

CMO: context–mechanism–outcome
IKT: integrated knowledge translation
KT: knowledge translation
STEPP: Scoresheet for Tangible Effects of Patient Participation
